# Atypical desmoplastic small round cell tumor (DSRCT) mimicking muscle-invasive bladder cancer (MIBC) in a child: Surgical approach using ileal diversion

**DOI:** 10.1016/j.radcr.2026.06.024

**Published:** 2026-07-07

**Authors:** Leecarlo Millano Lumban Gaol, Yuliana Saraswati Kusuma Prabaningrum, Andy, Aulia Fitri Swity, Vinesia Lestari Riddi, Yohanes Firmansyah, Bryan Anna Wijaya, Fiona Valencia Setiawan, Zaqiah Sri Rahayu

**Affiliations:** a Department of Surgery, Pediatric Surgery Division, Tarakan General Hospital, Central Jakarta, Indonesia; b Department of Surgery, Urology Division, Tarakan General Hospital, Central Jakarta, Indonesia; c Department of Pediatrics and Child Health, Tarakan General Hospital, Central Jakarta, Indonesia; d Department of Anatomical Pathology, National Cancer Center Dharmais Hospital, West Jakarta, Indonesia; e Faculty of Medicine, Krida Wacana Christian University, Jakarta, Indonesia; f Faculty of Medicine, Tarumanagara University, Jakarta, Indonesia; g Faculty of Medicine, Pembangunan Nasional “Veteran” University, Jakarta, Indonesia

**Keywords:** Desmoplastic small round cell tumor, Ileal conduit urinary diversion, Muscle-invasive bladder cancer mimic, Pediatric bladder cancer involvement, Radical cystectomy

## Abstract

We report an aggressive rare case of desmoplastic small round cell tumor (DSRCT) mimicking muscle-invasive bladder cancer (MIBC) in a 14-month-old boy. The patient underwent radical cystectomy with ileal diversion after multidisciplinary assessment. Histopathology confirmed DSRCT. This case highlights diagnostic challenges and the role of surgical management in pediatric bladder-involved DSRCT.

## Introduction

Bladder cancer (BCa), a common urinary malignancy, classified as muscle-invasive (MIBC) or non-muscle-invasive (NMIBC) based on the tumor invasion depth [1]. While most cases of BCa present as NMIBC, about 30% will progress to MIBC over time [2]. The incidence of bladder cancer is approximately 9 and 2 cases per 100,000 population in men and women, respectively (based on 2008 data). While bladder cancer predominantly affects adults, with a peak incidence in the sixth decade of life, it remains rare in the pediatric population, with only a few reported cases [3].

Desmoplastic small round cell tumor (DSRCT) is an aggressive mesenchymal malignancy of uncertain origin, primarily affecting males aged 5-50 years (mean: 22 years). It typically arises in the abdominal or pelvic cavity, with an incidence of only 0.2 per million [[4], [5], [6]]. DSRCT most often involves serosal surfaces, including the peritoneum, pleura, and tunica vaginalis. Involvement of the urogenital tract, particularly in children, is exceptionally uncommon [7].

While radical cystectomy (RC) with urinary diversion remains the gold standard for MIBC management, DSRCT requires a multimodal approach involving chemotherapy, radiotherapy, and aggressive cytoreductive surgery [4,[8], [9]].

Here, we report a rare case of pediatric DSRCT mimicking MIBC, in which radical cystectomy with ileal diversion was chosen as the most appropriate treatment after multidisciplinary consideration.

## Case presentation

A 14-month-old boy presented with urinary retention, progressive abdominal distension, and decreased appetite. He had previously been hospitalized for 10 days and underwent urinary catheterization. Persistent hematuria and suspicion of an aggressive pelvic mass prompted referral to our tertiary center. On admission, he appeared lethargic with lower limb edema and a palpable, firm suprapubic mass. Detailed clinical and laboratory findings are summarized in Fig. 1.

**Fig. 1:**
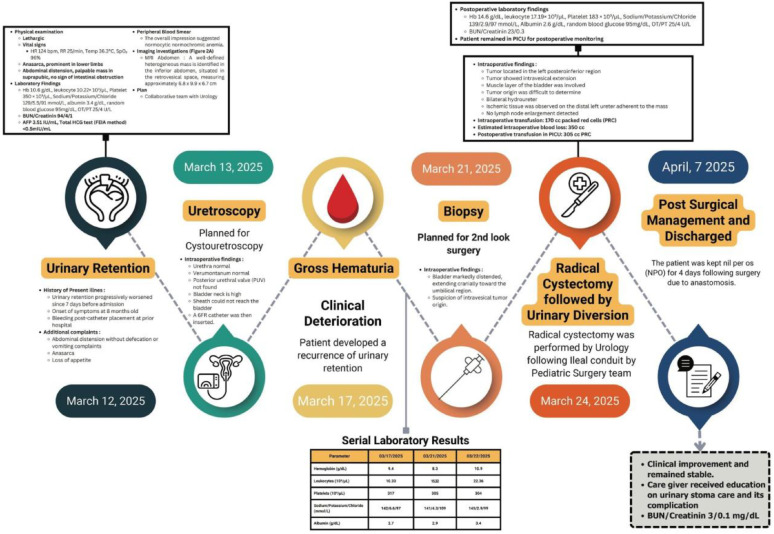
Clinical and investigation summary during the first admission at our institution.

### Birth and maternal history

He was the second child of a G3P2 mother, delivered at term (40 weeks) via spontaneous vaginal delivery. The pregnancy initially involved twins, with one fetus non-viable. Antenatal ultrasound incidentally detected a multilobulated cyst in the maternal left ovary. There was no maternal comorbidity or family history of malignancy, although several paternal uncles had kidney disease. The father was an active smoker, and the family resided in a suburban area.

### Imaging investigations

Initial evaluation was performed with a CT scan at the referring hospital, followed by an abdominal MRI at our institution for further assessment. The CT scan demonstrated a large, solid, lobulated pelvic mass measuring approximately 71 × 74 × 85 mm, extending from the lower lumbar spine to the paravertebral region and anterior abdominal wall, accompanied by right pleural effusion.

MRI revealed a well-defined heterogeneous mass in the lower abdomen, located posterior to the urinary bladder (retrovesical region), measuring 6.8 × 9.9 × 6.7 cm, with bilateral hydronephrosis likely due to ureteral compression by the mass (Fig. 2A).

**Fig. 2:**
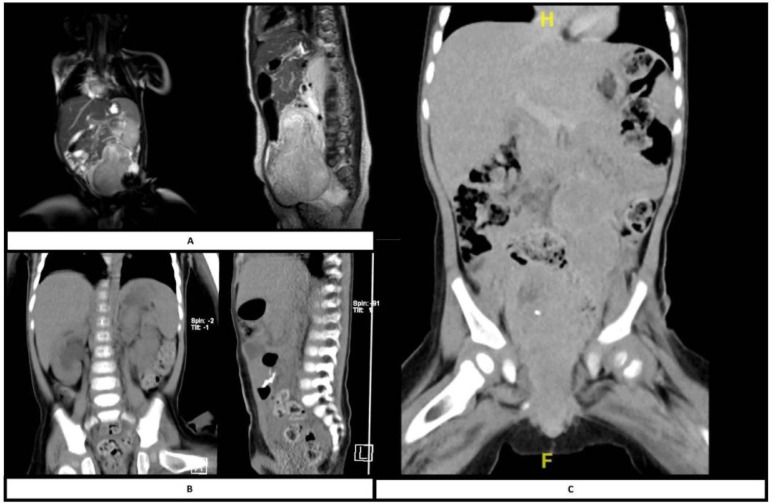
(A) Abdominal MRI showing a large pelvic mass at initial evaluation. (B) Non-contrast abdominal CT scan (first evaluation), demonstrating bilateral hydronephrosis. (C) Follow-up contrast-enhanced abdominal CT scan showing multiple lobulated masses with necrotic components.

Two weeks post-radical cystectomy and ileal conduit diversion, a non-contrast CT scan showed persistent bilateral hydronephrosis without evidence of gastric or intestinal dilatation, focal lesions, calcifications, ascites, or pleural effusion (Fig. 2B).

Subsequently, a contrast-enhanced CT scan was performed to reassess disease progression. Multiple heterogeneous, lobulated masses with necrotic foci throughout the mesentery, the largest measuring approximately 8.3 cm in the minor pelvis (Fig. 2C). This evaluation was performed seven weeks after radical cystectomy with ileal conduit diversion.

### Surgical findings and post-operative management

The patient underwent four separate surgical procedures. Initial urethroscopy revealed outlet obstruction suggestive by the tumor, necessitating catheterization. Exploratory laparotomy with biopsy was followed by radical cystectomy and ileal conduit diversion. Upon readmission, nephrostomy was performed to manage pyonephrosis.

The perioperative course is summarized in Fig. 1, and the urinary diversion technique is illustrated in Fig. 3 [10,11].

**Fig. 3:**
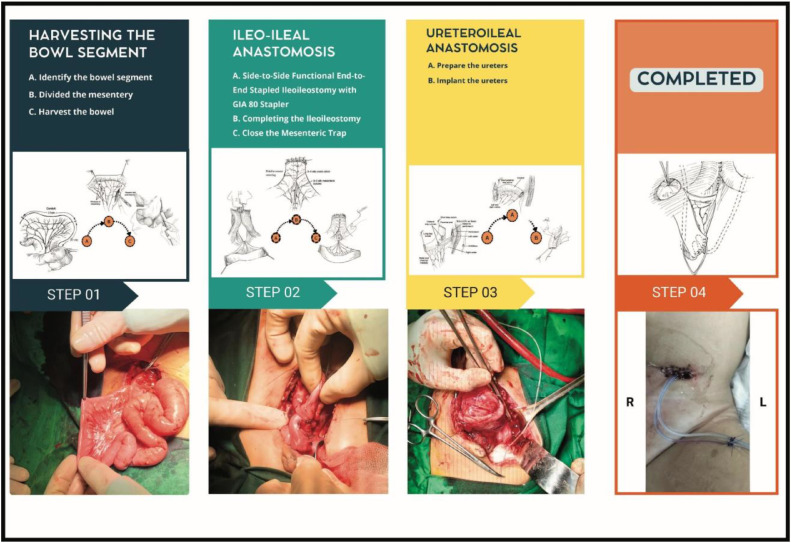
Schematic of the ileal conduit procedure. Modified from: Scherr DS, Barocas DA. Hinman's Atlas of Urologic Surgery (2012), pp. 615-627, and intraoperative documentation, Tarakan General Hospital, 2025.

### Histopathology and immunohistochemistry (IHC)

Gross examination revealed an irregular, firm brown bladder mass measuring 14 × 8 × 5.5 cm, with solid whitish areas and necrotic foci infiltrating the perivesical fat. The bladder lumen showed a 2-cm cavity filled with brown tumor material. A small firm tissue fragment (1 × 0.5 × 0.5 cm) from the left ureter showed no tumor involvement.

Microscopically, the lesion consisted of spindle to polygonal malignant cells within a myxoid stroma, displaying pleomorphic hyperchromatic nuclei, frequent mitoses, necrosis, and lymphovascular invasion (See Fig. 4A). Occasional rhabdoid-like cells were identified. The initial histologic impression favored embryonal rhabdomyosarcoma; however, immunohistochemistry (IHC) confirmed DSRCT, with positive staining for CD99 and desmin and negative for MyoD1, SMA, and EMA (See Fig. 4B). The IHC confirmation was finalized one month after the initial pathology report.

**Fig. 4:**
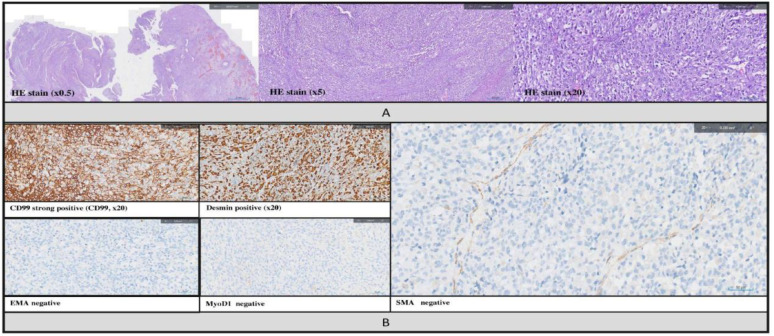
Histopathology and Immunohistochemistry (IHC). (A) Histology of tumor with HE stains. (B) Immunohistochemistry.

### Clinical deterioration and outcome

The patient was discharged on April 7, 2025, with clinical improvement. A follow-up CT on May 15 showed tumor recurrence, but the patient was lost to follow-up. His condition worsened three days before readmission, with signs of intestinal obstruction. Chemotherapy (vincristine, mesna–cyclophosphamide, doxorubicin) was started at 70% dose nearly three weeks after readmission, despite clinical instability. Timeline and outcomes are summarized in Fig. 5.

**Fig. 5:**
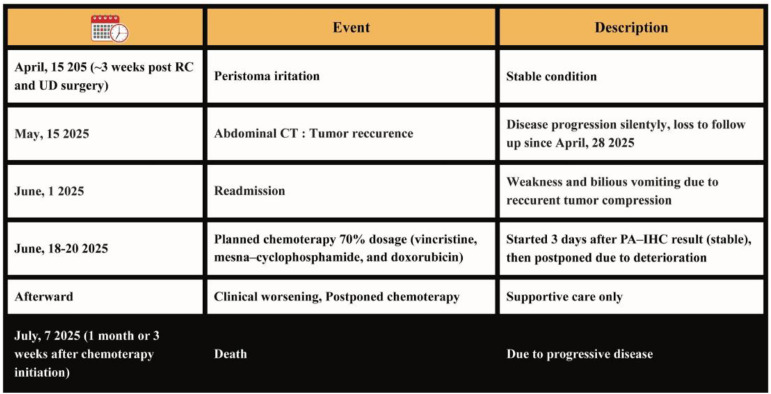
Timeline and outcomes post discharged.

## Discussion

DSRCT typically presents as multiple intra-abdominal masses with a multiphenotypic immunohistochemical profile [6], often infiltrating adjacent structures such as the omentum, mesentery, and serosal surfaces (peritoneum, pleura, scrotum) [4,7].

This tumor is characterized by a reciprocal translocation [t(11;22)(q13;q12)] resulting in fusion of the Ewing sarcome gene (EWSR1) on chromosome 22 with the Wilms tumor gene (WT1) on chromosome 11 [4,6,7,9]. The EWSR1–WT1 fusion gene serves as a defining molecular hallmark of DSRCT, encoding an aberrant transcription factor in which the WT1 component, normally involved in urogenital development, becomes oncogenic. This discovery has improved diagnostic precision and identified potential therapeutic targets, although additional factors likely contribute to tumorigenesis [4].

In the present case, the patient demonstrated abdominal compartment syndrome and gross hematuria caused by tumor compression and progression. Although hematuria suggests urologic malignancy in up to 25% of cases [12], bladder cancer is exceptionally uncommon in children [2,3,12]. Due to its atypical presentation, bladder-involved DSRCT may be misdiagnosed as more common bladder malignancies, including MIBC.

Diagnosis DSRCT relies on imaging and biopsy, as conventional modalities may underestimate disease extent, emphasizing the need for clinicopathologic correlation [4,6,7]. Extra-abdominal DSRCT involving the urogenital tract is exceedingly rare and may radiologically mimic transitional or urachal carcinoma, complicating diagnosis [7]. The limited number of cases and absence of a standardized staging system make management challenging. Current advances in DSRCT management explore hyperthermic intraperitoneal chemotherapy (HIPEC), adjuvant whole abdomen radiotherapy (WART), and immunotherapy targeting androgen receptor pathways [4,5,9]. Nevertheless, despite current multimodal strategies—surgery, chemotherapy, and radiotherapy—outcomes remain poor, with high recurrence rates and a 5-year overall survival of only 10%-15% [5].

While DSRCT is managed with multimodal therapy, surgery plays a key role in achieving optimal cytoreduction [4,6]. Chemotherapy is often administered as first-line treatment, followed by additional multimodal approaches, with surgery performed after significant tumor reduction—typically around six months post-chemotherapy [[4], [5], [6], [7],^9^]. However, the present case posed both diagnostic and therapeutic challenges.

In rare instances of bladder involvement, radical cystectomy may be required to achieve local control, as reported in similar case [7]. To our knowledge, this is among the few reported pediatric DSRCT cases involving the urinary bladder requiring radical cystectomy. This case thus provides valuable insight into the limited literature describing such a therapeutic approach in DSRCT.

Recent advances in BCa management have promoted the use of bladder preservation therapy (BPT); however, BPT is appropriate only for highly selected cases with preserved bladder function and favorable tumor characteristics. In high-risk patients, its efficacy markedly declines, and delayed radical surgery may worsen prognosis [1]. Therefore, radical cystectomy (RC) remains the mainstay for selected NMIBC, MIBC, and even metastatic cases [1,8].

Given this patient’s invasive disease, ureteral obstruction, and absence of distant metastasis, radical cystectomy followed by urinary diversion (UD) was considered the most appropriate approach after multidisciplinary discussion.

UD is indicated for malignancies or severe bladder dysfunction causing obstruction, bleeding, or upper tract damage [[13], [14], [15], [16]]. It may be either continent or incontinent, depending on patient condition and feasibility. Continent urinary diversion is designed to keep the continence system intact by providing a channel that can be catheterized for emptying [14,15]. Meanwhile, incontinent urinary diversion is recommended for patients who either cannot or choose not to perform clean intermittent catheterization (CIC), as well as for those experiencing upper urinary tract deterioration [15]. In pediatric patients, ileal conduit diversion remains common due to simplicity and reliability [14,15]. Literature recommends using a 15-20 cm ileal segment to minimize urine exposure and absorption [[13], [14], [15], [16]]; in this case, a 10 cm segment was used, placed in the left lower quadrant to accommodate preserved organs.

Choosing a UD is often debated and depends on several important factors [14]. Although some studies suggest lower complication rates with continent UD, long-term outcomes appear comparable [[15], [16], [17]].

At two months postoperatively, the patient maintained normal renal and electrolyte function without urinary retention or stenosis. However, he later developed pyelonephritis, recurrent abdominal distension, and intestinal obstruction, likely from tumor recurrence. It remains uncertain whether these complications were postoperative or due to tumor aggressiveness.

## Conclusion

This case underscores the importance of early recognition and multidisciplinary management of pediatric bladder malignancies such as DSRCT. While HIPEC may offer prognostic benefits, its role remains under investigation. RC with UD, combined with multimodal therapy, can provide local control and improve quality of life. Early recognition and timely referral are essential for improving outcome in pediatric DSRCT.

## Limitations

This case has several limitations. Only a contrast-enhanced abdominal CT scan was performed, without a dedicated CT urogram, which may have limited detailed assessment of the upper urinary tract. The histopathological confirmation process was delayed due to the need for expert review, resulting in postponed chemotherapy initiation. Molecular confirmation of the EWS–WT1 fusion gene and hyperthermic intraperitoneal chemotherapy (HIPEC) could not be performed due to limited resources. Additionally, the patient’s unstable clinical condition and loss to follow-up after radiologic recurrence hindered. Nevertheless, this report adds valuable insight into surgical decision-making in rare pediatric DSRCT.

## Authorship and copyright statement

All figures, illustrations, and other visual materials presented in this manuscript were independently conceived, produced, and finalized by the authors. No third-party copyrighted content was incorporated, thereby ensuring complete originality and full compliance with applicable intellectual property regulations.

## Ethical approval

Ethical approval was not required for this study.

## Author contributions

**Leecarlo Millano Lumban Gaol:** Conceptualization, Supervision, Validation, Funding acquisition, Investigation, Writing – review & editing. **Yuliana Saraswati Kusuma Prabaningrum:** Data curation, Investigation, Writing – original draft, Writing – review & editing. **Andy:** Investigation, Resources. **Aulia Fitri Swity:** Investigation, Resources. **Vinesia Lestari Riddi:** Investigation, Validation. **Yohanes Firmansyah:** Data curation, Investigation, Visualization. **Bryan Anna Wijaya:** Data curation, Visualization. **Fiona Valencia Setiawan:** Data curation. **Zaqiah Sri Rahayu:** Writing – review & editing.

## Patient consent

A written informed consent was obtained from the patient for the publication of this case report.

## References

[1] Li F., Zhang Y., Wang Y. (2025). Advances in bladder preservation therapy for muscle-invasive bladder cancer. Front Oncol.

[2] Aykan S., Yuruk E., Tuken M., Temiz M.Z., Ozsoy S. (2015). Rare but lethal disease of childhood: metastatic, muscle invasive bladder cancer. Pediatr Rep.

[3] Polat H., Utangac M.M., Gulpinar M.T., Cift A., Erdogdu I.H., Turkcu G. (2016). Urothelial neoplasm of the bladder in childhood and adolescence: a rare disease. Int Braz J Urol.

[4] Gawash A., Simonetti A., Goodwin B.J., O’Neill A.B. (2025). Desmoplastic small round cell tumor: an update of current management practices. J Egypt Natl Canc Inst.

[5] Offenbacher R., Lazar P., Fabish L., Fox J., Lee A., Loeb D.M. (2025). Strategies for the treatment of desmoplastic small round cell tumor: A case series. Pediatr Blood Cancer.

[6] Hayes-Jordan A., LaQuaglia M.P., Modak S. (2016). Management of desmoplastic small round cell tumor. Semin Pediatr Surg.

[7] Diao B., Li F., Li G., Chen Z., Bi C. (2010). Primary desmoplastic small round cell tumor of urinary bladder. J Clin Oncol.

[8] Aminoltejari K., Black P.C. (2020). Radical cystectomy: a review of techniques, developments and controversies. Transl Androl Urol.

[9] Yamauchi K., Owari M., Kimura K., Ishii T., Wakasa T., Yonekura T. (2023). Desmoplastic small round cell tumor: a case report and literature review. J Pediatr Surg Case Rep.

[10] Scherr D.S., Barocas D.A., Smith J.A., Howards S.S., Preminger G.M., Dmochowski R.R. (2012). Hinman's atlas of urologic surgery.

[11] Gaol LML. (2025). Personal documentation Ileal conduit procedure.

[12] Boorjian S.A., Raman J.D., Barocas D.A., Wein A.J., Kavoussi L.R., Partin A.W., Peters C.A. (2020). Campbell-Walsh urology..

[13] Konety B.R., Barbour S., Carroll P.R., Wein A.J., Kavoussi L.R., Partin A.W., Peters C.A. (2019). Smith & tanagho’s general urology.

[14] Browning J.D., Stephany H.A. (2018). Urinary diversion: indications and options. Urol Clin North Am.

[15] Stein R., Zahn K., Huck N. (2019). Current indications and techniques for the use of bowel segments in pediatric urinary tract reconstruction. Front Pediatr.

[16] Skinner E.C., Daneshmand S., Wein A.J., Kavoussi L.R., Partin A.W., Peters C.A. (2020). Campbell-Walsh urology.

[17] Chiacchio G., Mastroianni R., Simone G. (2024). Continent or incontinent urinary diversion: that is the question. Ann Surg Oncol.

